# A streamlined tethered chromosome conformation capture protocol

**DOI:** 10.1186/s12864-016-2596-3

**Published:** 2016-04-01

**Authors:** Idan Gabdank, Sreejith Ramakrishnan, Anne M. Villeneuve, Andrew Z. Fire

**Affiliations:** Department of Genetics, Stanford University School of Medicine, Stanford, CA 94304 USA; Departments of Developmental Biology and Genetics, Stanford University School of Medicine, Stanford, California 94304 USA; Departments of Pathology and Genetics, Stanford University School of Medicine, Stanford, California 94304 USA

**Keywords:** Hi-C, TCC, Chromatin, Conformation, *Caenorhabditis elegans*, Genome

## Abstract

**Background:**

Identification of locus-locus contacts at the chromatin level provides a valuable foundation for understanding of nuclear architecture and function and a valuable tool for inferring long-range linkage relationships. As one approach to this, chromatin conformation capture-based techniques allow creation of genome spatial organization maps. While such approaches have been available for some time, methodological advances will be of considerable use in minimizing both time and input material required for successful application.

**Results:**

Here we report a modified tethered conformation capture protocol that utilizes a series of rapid and efficient molecular manipulations. We applied the method to *Caenorhabditis elegans*, obtaining chromatin interaction maps that provide a sequence-anchored delineation of salient aspects of *Caenorhabditis elegans* chromosome structure, demonstrating a high level of consistency in overall chromosome organization between biological samples collected under different conditions. In addition to the application of the method to defining nuclear architecture, we found the resulting chromatin interaction maps to be of sufficient resolution and sensitivity to enable detection of large-scale structural variants such as inversions or translocations.

**Conclusion:**

Our streamlined protocol provides an accelerated, robust, and broadly applicable means of generating chromatin spatial organization maps and detecting genome rearrangements without a need for cellular or chromatin fractionation.

**Electronic supplementary material:**

The online version of this article (doi:10.1186/s12864-016-2596-3) contains supplementary material, which is available to authorized users.

## Background

The spatial organization of the eukaryotic genome is now accessible through techniques involving massive parallel high-throughput sequencing ([1–3]). An understanding of how chromosomes fold can provide insight into complex relationships between chromatin structure, genetic activity and functional state of the cell ([4, 5]). In addition, genome wide chromatin interaction data sets can reveal long-range information about the grouping and linear organization of sequences along entire chromosomes, enabling high quality chromosome-scale de novo genome assembly [6].

Chromosome conformation capture (3C)-based techniques have emerged as powerful tools for mapping chromatin contacts ([1, 3, 7–13]). One recently-described technique, tethered conformation capture (TCC) [14], was developed to improve signal to noise ratio over previously published techniques, allowing in-depth analysis of both intra and inter-chromosomal contacts. 3C-based techniques use proximity ligation and massively parallel sequencing to probe the three-dimensional architecture of chromosomes within the nucleus, with closely interacting regions captured via the ligation step and identified through sequence analysis. In the resulting data sets, the probability of intra-chromosomal contacts is on average much higher than that of inter-chromosomal contacts, as expected if chromosomal territories are at least partially distinct. These data have supported a model in which chromosomal territories are indeed distinct entities: although the probability of interaction decays with linear distance, even loci separated by megabases on the same chromosome are more likely to interact than loci on different chromosomes.

We have developed a rapid tethered conformation capture (RTCC) technique to allow fast application of a TCC-based protocol in *C. elegans*. Our method allows detection of chromatin contacts using unfractionated whole tissues or whole organisms as starting material, avoiding extensive cellular and molecular fractionation steps. Applied to *C. elegans*, this protocol illuminates both the large scale structural partitioning (e.g., [15–21]) and fine resolution genome architecture dynamics (i.e. Crane et al. [22]).

## Results and discussion

### Detecting genome-wide chromatin contacts using RTCC

RTCC differs from previous chromosome conformation capture protocols in two key respects: (i) the lack of a need for cellular or chromatin fractionation, (ii) the application of efficient transposon tagging approach toward capture of potential ligation junctions.

**Fig. 1:**
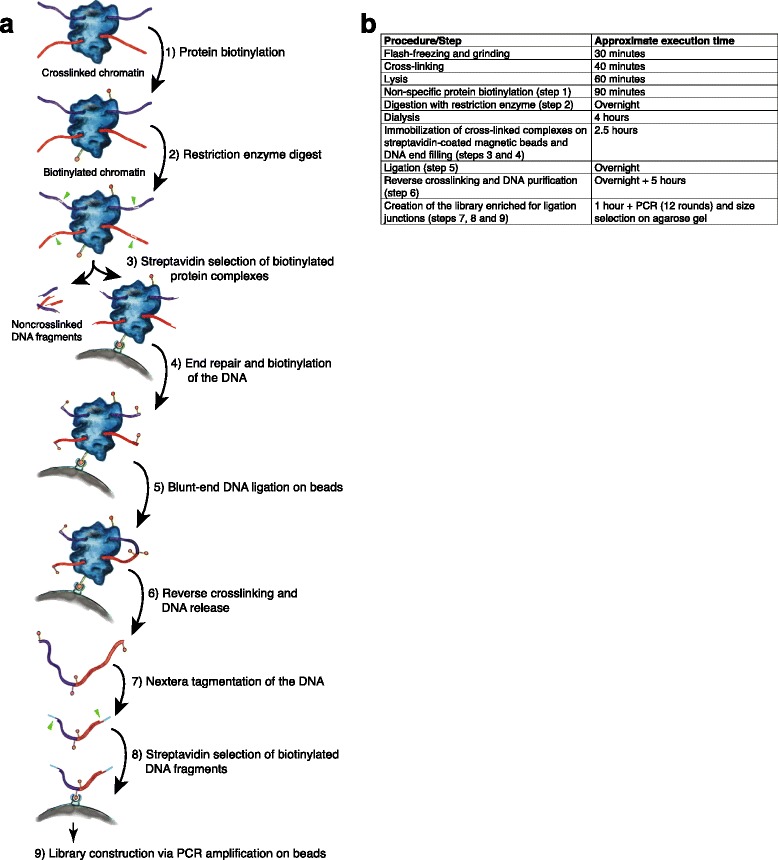
Overview of RTCC protocol. **a** Diagram shows a schematic description of steps from a crude tissue homogenate to a proximity sequencing library (details provided in the Methods section). For our studies (using *C. elegans*), animals flash-frozen in liquid nitrogen were finely ground using either mortar and pestle or using an electric drill with “Cellcrusher” drill-bit and “Cellcrusher” base held at liquid nitrogen temperature and treated with formaldehyde to covalently cross-link proteins to each other and to DNA (red and purple strands, threaded through the blue amorphous complex, representing proteins). (1) Chromatin is solubilized with detergent and proteins were non-specifically biotinylated (orange balls on sticks). (2) DNA was digested with a restriction enzyme that generates 5’ overhangs. (3) Cross-linked complexes were immobilized at a very low density on the surface of streptavidin-coated magnetic beads (grey color arc) through the biotinylated proteins, while the non-cross-linked DNA fragments were removed. (4) 5′ overhangs were filled in using DNA polymerase and a nucleotide mixture containing biotin-14-dCTP (orange balls on sticks) to generate blunt ends. (5) Blunt DNA ends were ligated. (6) Cross-linking was reversed and DNA was purified. (7) The DNA was fragmented and tagged (light blue strands) using Nextera tagmentase. (8) DNA fragments containing biotinylated CTP were selected on streptavidin-coated beads. This selects for ligation junctions and DNA molecules biotinylated at their terminus. (9) A Sequencing library was generated via PCR using the Nextera [http://www.illumina.com/products/nextera_dna_library_prep_kit.html] adaptors introduced at step 7. This amplification step should provide a substantial enrichment for ligation junctions, since molecules that were biotinylated solely on their termini would carry a Nextera adaptor only on one side. **b** RTCC protocol timeline

Figure 1b outlines the RTCC protocol with approximations of time for each step. Of note are the avoidance of cellular or chromatin fractionation steps in standard Hi-C, and the adaptation of the protocol for low input volumes in sequencing library preparation by adapting Nextera tagmentase [23] to sequencing of biotin-labeled junctions.

To preserve native features of genomic organization, whole worms were flash frozen and finely ground under liquid nitrogen. These frozen samples were quickly resuspended and subjected to formaldehyde treatment to chemically crosslink DNA and proteins. This material could then be used directly for molecular manipulations and analysis (Fig. 1), avoiding any need for an intervening nuclear or chromatin isolation step. In the cross-linked lysates, DNA was digested with a restriction enzyme, proteins were biotinylated non-specifically (cysteine biotinylation) [14], protein-DNA cross-linked complexes were immobilized at a low surface density on streptavidin-coated beads, and physically juxtaposed free DNA ends were filled in with a nucleotide mixture containing biotin-14-dCTP. Ligation of ends was then performed while fragments remained tethered to the surface of the beads. Following reversal of crosslinking and release of DNA, the Nextera tagmentation (transposon tagging/fragmentation) protocol was used to fragment the DNA and add linkers for sequencing in a single step [23]. The ligation junctions were purified by selection for biotinylated DNA fragments, which were subjected to massively parallel sequencing. Mapping of the sequenced junctions allowed detection of genomic locations for pairs of contacting loci.

**Fig. 2:**
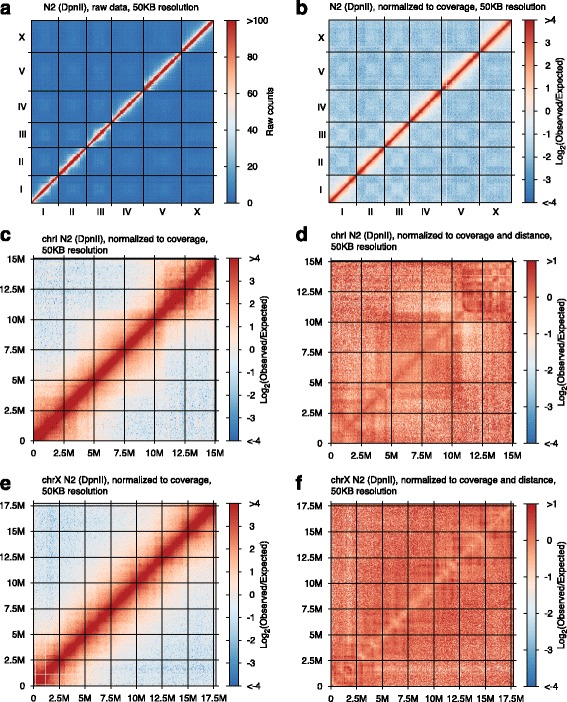
Chromatin interaction intensity maps. **a** Heat map showing raw counts of observed chromatin contacts on a genome-wide scale with 50KB bins (data from wild type N2 young adults). **b** Binned chromatin interaction map for wild type N2 young adults displayed with color representing the Log_2_ of the observed/expected ratio for each 50KB bin pair. **c** Magnified Log_2_ plot as in B, but focused just on chromosome I. **d** A further normalization of the plot in Panel C in which the interaction level for each combination of 50KB intervals is normalized to other pairs of intervals separated by a similar distance (using HOMER software [40]). **e** Log_2_ of the observed/expected ratio of interaction frequency (similar to panel C) for the X chromosome. **f** Coverage and distance normalized interaction plot (similar to panel D) for the X chromosome

We have applied RTCC to a variety of different tissue samples derived from *C. elegans*, using the DpnII, AvaII, and HindIII restriction enzymes, which cut respectively with 4, 4.5, and 6 base recognition specificities (appropriate restriction enzymes for this protocol must have the properties of leaving a 5' overhang, and of having an overhang for which one base is a “C”.) Exemplary chromatin interaction intensity maps are shown in Fig. 2. We then applied several approaches in evaluating the consistency and accuracy of this large-scale dataset.

First, we evaluated the consistency of results from this approach by comparing results from different experimental replicates. The replicates were carried out with slight deviations in the underlying protocol, distinct stages and tissue distributions, and different choices of restriction enzymes; hence their consistency becomes a test for both biological and technical reproducibility. Indeed we observed that experimental replicates (Additional file 1: Figure S1) were highly correlated (Pearson’s correlation coefficients vary between 0.76 and 0.97 for 50KB binned data).

Second we compared the chromatin interaction matrices obtained with RTCC with a recent Hi-C analysis performed using *C. elegans* embryos, acquired by Crane et al. [22] towards understanding of X chromosome topology remodeling during dosage compensation. This comparison likewise demonstrates experiment-to-experiment correlation, both by inspection (Additional file 2: Figure S2) and from calculating a formal Pearson correlation coefficient (>0.64).

Further validation of the RTCC data that we obtained comes from analysis of distances between interacting regions captured by RTCC ligation. With any proximity ligation protocol (even when optimized), we expect a subset of artefactual ligation events that will join DNA segments with no true association (in some cases, even DNA sequences from two different cells). Such events might be expected to lack a clear dependence on inter-locus distance, while bone-fide contacts that were captured from a physiological chromosome configuration would be expected to be much more frequent for closely linked loci.

**Fig. 3:**
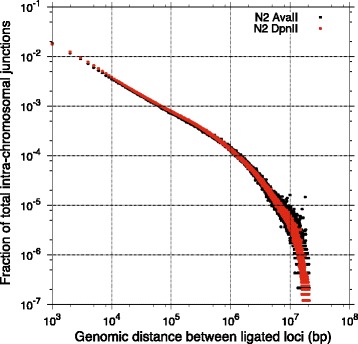
Interaction frequency decay as function of the distance between interacting loci. The genome was divided into 1KB non-overlapping intervals and intra-chromosomal contacts between these intervals were counted to produce the plotted profile. In the chart we are plotting the fraction of the total intra-chromosomal junctions detected within 1000 bp of a given genomic distance

**Fig. 4:**
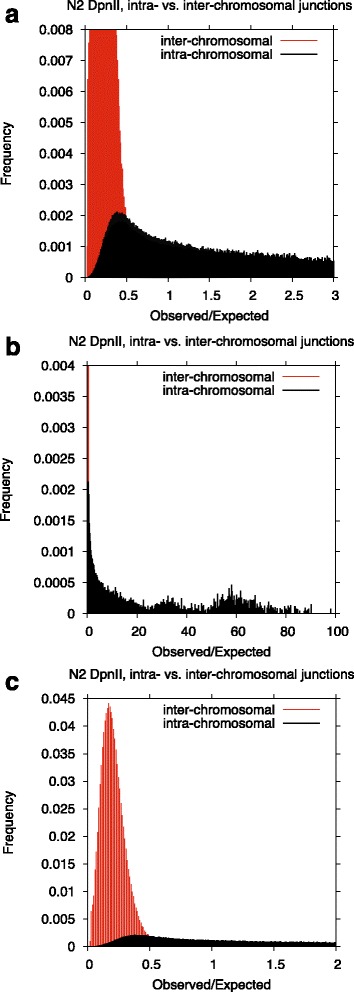
Differences in prevalence of intra- and inter-chromosomal contacts. Contacts detected in aggregated N2 DpnII experiments (total of ~18M junctions) were used to construct an interaction frequency matrix with resolution of 50KB. **a**, **b**, **c** show sections of this chart under different magnifications. For each square in the matrix we calculated an expected number of contacts (based on the product of sequence read coverage for the two regions amongst all “junctional” reads) and compared these with the quantity of the observed “junctional” reads between the two indicated regions. We have plotted the frequencies of observed/expected ratios in intra- and inter-chromosomal junctions for each bin of width 0.001 in observed/expected value. Junctions mapping to ribosomal RNA sequence on chromosome I were excluded from the calculation

Our data shows an expected and dramatic inverse association between the distance between the interacting loci and the number of intra-chromosomal contacts (Fig. 3). Dependence of the chromatin contacts on the distance between interacting regions is consistent with DNA polymer-like behavior in which three-dimensional distance between loci increases with increasing genomic distance [1]. Analysis of frequencies of different types of contacts shows greater frequency of intra-chromosomal contacts than inter-chromosomal (Fig. 4) supporting the idea of chromosome territoriality [24].

### Chromatin interaction maps

Using the interaction information we obtained from the mapping of the sequenced DNA library (see Methods), we created a genome-wide raw chromatin interaction matrix. In order to create this matrix, the genome was divided into segments, the size of which depends on the depth of sequencing analysis and level of resolution required (Fig. 2 shows a series of such maps with a 50KB segment length). Each cell in the matrix *m*_*i,j*_ corresponds to the number of contacts (proximity ligation products) between segment *i* and segment *j* of the genome. The interaction matrix can be depicted visually with a heat map, in which the color intensity correlates with contact frequency (Fig. 2a).

A whole genome raw contacts map for *C. elegans* shows 6 distinct squares aligned on the diagonal, each corresponding to an individual chromosome, supporting the idea that the probability of intra-chromosomal contacts is on average much higher than that of inter-chromosomal contacts and that chromosomes occupy distinct territories.

The whole genome chromatin interaction matrix was normalized using the expected number of contacts assuming each region has an equal chance of interacting with every other region in the genome, essentially normalizing to read coverage at each region. The resulting normalized whole genome matrix, presented as a heat map in Fig. 2b, exhibits more clear separation into 6 chromosomes and shows some of the intra-chromosomal organization features as well.

Zooming in to the single chromosome level (Fig. 2 c-f) allows study of intra-chromosomal structural organization features ([25, 26]). Tendencies for arm-arm and center-center interaction on the autosomes are evident from two dimensional heat maps that associate each combination of genome positions (x and y) with a color indicating the degree of over- or under-representation in the population of novel junctions. This observation is indicative of a physical basis for the observation that *C. elegans* autosomes show distinct features in arm and center regions (although both can house active genes, there is a tendency for constraint of contacts between chromosome arms and centers). These findings are consistent with results from linear analyses of chromosomal features ([15–21]), from cytological studies [27] and from another recently communicated chromosome capture analysis by Crane et al. [22]. Additional file 3: Figure S3 shows a remarkable association between center-arm positioning within each autosome for our data and data from Crane et al. [22]. One physical correlate of the association is suggested by alignment with a dataset derived from immunoprecipitation [28] using an antibody against the nuclear envelope component LEM-2 (Additional file 3: Figure S3, cyan). As noted also by Crane et al. [22] our data point to a more complex organization on the X chromosome, with evidence for a domain organization that differs from the end-center-end organization observed on the five autosomes.

Several years ago, it was noted that a subset of DNA segments on *C. elegans* autosomal arms exhibit a strongly periodic sequence character, with phased runs of A and T residues. Known as the “PATCs” regions, these genomic features are characteristic of introns and other noncoding sequences for a subset of genes expressed in the *C. elegans* germ line [29]. We found that PATC character (for which we use the quantitative measure defined in [29]) was strongly associated with partitioning of contacts between central and arm regions of chromosomes (Table 1) This tendency appears to be a general property of this unusual genomic partition, as all five autosomes show similar arm-association-with-arm enrichment on both arms (Table 1). This correspondence suggests a strong tendency for PATC-rich regions to inhabit their own subdomain of the connectivity network, and is consistent with proposed roles of such extended DNA sequence features in long range chromosomal organization [29].

**Table 1:** Correlation between Phased A_n_/T_n_ Cluster (PATC) content and preference for interaction with chromosome arms

Correlations for arms regions ONLY	chrI	chrII	chrIII	chrIV	chrV	chrX
log_2_(arms/centers) vs. PATCs 25KB Pearson R values	0.38	0.37	0.17	0.44	0.26	0.51
log_2_(arms/centers) vs. PATCs 25KB Spearman R values	0.36	0.50	0.18	0.45	0.48	0.49
log_2_(arms/centers) vs. PATCs 50KB Pearson R values	0.50	0.58	0.25	0.54	0.30	0.63
log_2_(arms/centers) vs. PATCs 50KB Spearman R values	0.46	0.60	0.25	0.55	0.58	0.61

To measure correlation of inter-chromosomal contacts between chromosomal arms regions (defined according to [20]) and PATC enrichment we divided the genome into 25KB (50KB) non-overlapping intervals. For each interval we measured the enrichment for PATC by counting the number of locations with high PATC score (defined as regions with a score of >55 using the algorithm of [29]). In order to measure the level of enrichment of inter-chromosomal arm-arm contacts over the arm-centers contacts we calculate the number of inter-chromosomal arm-arm contacts and number of inter-chromosomal arm-center contacts for every interval. Log_2_ value of the enrichment for inter-chromosomal arm-arm contacts is used to calculate correlation with PATC enrichment in different genomic regions

### Modeling-based evaluation suggests utility of RTCC in identification of chromosome structural variation

**Fig. 5:**
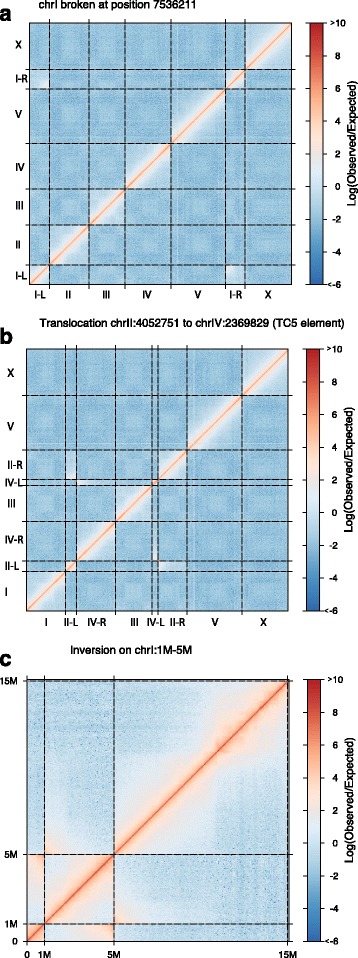
Detection of simulated structural variants by RTCC. This figure shows an analytical evaluation of feasibility for using RTCC data to detect large scale structural variations in the *C. elegans* genome. For this analysis, the N2 reference genome was computationally modified to simulate “model reference genomes” that differ structurally from the normal *C. elegans* genome. We then analyzed the data from N2 (DpnII) experiments as in Fig. 2, aligning the reads to the indicated simulated reference genome and using a 50KB window size as above. In each case, the analysis yields a footprint characteristic of the simulated rearrangement. In (**a**), we simulated a two-chromosome fusion: we generated a model reference genome in which chrI was artificially separated into 2 parts (I-L and I-R), each 7.53MB long. Execution of our analysis pipeline resulted in visible evidence for a high level of contacts between the artificially created right tip of I-L and the artificially created left tip of I-R of the model reference genome, which are “fused” in the N2 genome. In (**b**), we simulated a reciprocal translocation by creating a model reference genome in which segments of chromosomes II and IV were virtually recombined. The simulated recombination was created in the middle of a TC5 transposable element (3171bp long) present in multiple copies in the genome to simulate a rearrangement that would have presented detection challenges by standard methods. The “translocation” in the N2 data (relative to the model reference genome) is evident on the plot, by the distinct accentuation of contacts between II-L and II-R and IV-L and IV-R. In (**c**), we generated a model reference genome in which a large inversion (4MB in length) was virtually introduced on chromosome I. Evidence for inversion is visible on the plot

Information on genomic architecture from Hi-C and similar approaches has been useful in diverse genome-structure applications (e.g., [30, 31]). To evaluate the utility of RTCC in investigating structural variation, we produced three conceptual “model reference genomes” with substantial structural variation, one where the standard N2 reference genome is related by a chromosomal fusion, one where the N2 genome is related by a reciprocal translocation, and one where the N2 genome is related by a large inversion. To model “real world” structural variants, we constructed the translocation with breakpoints at a homologous point in an extended DNA repeat (the transposon Tn5). This rearrangement would have remained undetected with paired end or split read sequencing. As shown in Fig. 5 all of the simulated structural variants could be readily detected using RTCC analysis.

## Conclusions

In this study we modified and optimized TCC protocol to work with limited amounts of unfractionated tissue, avoiding subcellular fractionation and chromatin isolation.

The ability to measure proximity between genomic loci in linear space can be utilized both in structural studies of chromosomes and in applications such as genome assembly or phasing.

The RTCC protocol that we present here should provide an efficient and robust means of performing Hi-C experiments in general and in nematodes in particular. Our method allows detection of long-range contacts between genomic loci, supplying useful information to study three-dimensional organization of the genome. Further, Hi-C data sets generated using our RTCC protocol can provide a measurement of proximity between genomic elements in linear sense, allowing this knowledge to be used in bioinformatics applications such as LACHESIS for high quality genome assembly [6].

## Methods

### Nematode strains

Experiments were carried out with two *C. elegans* cultivars, each derived from the original wild type “N2” isolate used by Brenner [15], PD7052 is an N2 stock obtained from the Caenorhabditis Genetics Center (Minnesota, USA) in 2001, while PD1074 is a clonal isolate from the genomically defined N2-derivative VC2010 obtained from M. Edgley and colleagues [32]. Germ cell deficient animals were obtained as described below from the temperature sensitive mutant strain *glp-1(e2141ts)III* [33].

### L1 stage larvae starved and fed animals

To obtain synchronized wild-type L1 stage larvae, animals from N2 (PD7052) strain worms were grown in liquid culture in S-complete media [34] supplemented with *Escherichia coli* HB101 bacteria at 20 °C shaking at 180 rounds per minute. Embryos were obtained by standard bleaching protocol and hatched in sterile S-complete liquid media. The animals were starved for 24 h for population synchronization. Half of the synchronized starved L1 stage larvae animals were harvested and frozen in liquid nitrogen [“starved” sample], while the remainder [“fed” sample] were fed on HB101 bacteria for 3 h before harvesting and freezing in liquid nitrogen.

### Wild-type N2 and mutant *glp-1(e2142ts)III* young adult animals

Young adult populations were grown on enriched nematode growth medium plates with *Escherichia coli* OP50 bacteria [15] at 23 °C, the worm population was synchronized by standard bleaching protocol [15] and starvation for 24 h on unseeded nematode growth media plates at 16 °C. Synchronized L1 stage larvae animals were transferred to enriched nematode growth media seeded with *Escherichia coli* OP50 bacteria [15] and grown at 23 °C until reaching young adulthood.

In order to obtain germline-depleted populations, we used a temperature sensitive mutant, *glp-1(e2141ts)III,* which produces a gonad with approximately 20 sperm, in contrast to the thousand or more germ cells present in wild type animals [33, 35]. *glp-1(e2141ts)III* animals were grown on enriched nematode growth medium plates with *Escherichia coli* OP50 bacteria at permissive temperature (16 °C). The worm population was synchronized by standard bleaching protocol and starvation for 24 h on unseeded nematode growth media plates at 16 °C. Synchronized L1 stage larvae animals were transferred to enriched nematode growth media seeded with *Escherichia coli* OP50 bacteria at restrictive temperature (23 °C) [36]. The worms were grown at 23 °C until reaching young adulthood.

### RTCC Protocol

Animals were harvested by chilling on ice, centrifugation at 950g for two minutes and washing several times with cold M9. Pellets consisting each of approximately 100μl of closely packed worms were flash frozen in liquid nitrogen and stored in -80 °C. The flash frozen worm pellets were ground to fine powder in liquid nitrogen. The grinding was performed either using mortar and pestle or using an electric drill with “Cellcrusher” drill-bit and “Cellcrusher” base held at liquid nitrogen temperatures [http://cellcrusher.com/tissuepulverizer/, http://cellcrusher.com/drill-bit-2/]. The grinding was done for several minutes, until reaching a fine powder. The powder was stored at -80 °C.

Approximately 100 μl of liquid-nitrogen-ground tissue were used in L1 experiments, corresponding to ~2*10^6^ L1 animals, ~7.4*10^9^ haploid genomic copies, and yielding 5-10 μg of DNA in the final steps. For N2/glp-1 young adult experiments, we also used about 100 μl of worm powder, in this case corresponding to ~10^4^ adult animals, ~7*10^7^ haploid genomic copies [37] in N2 and about half of that in glp-1. In the final steps of the adult experiments we had 100-350 ng of DNA for Nextera tagmentation.

For both L1 and adult protocols, freeze-ground tissue was processed using procedures modified from [14]. Ground tissue was directly resuspended in 1 ml ice cold buffer A (15 mM Hepes-Na, pH 7.5, 60 mM KCl, 15 mM NaCl, 0.15 mM beta-mercaptoethanol, 0.15 mM spermine, 0.15 mM spermidine, 0.34 M sucrose) containing 1/100 dilution of HALT protease and phosphatase inhibitor cocktail (Thermo Scientific). 16 % Formaldehyde Solution (Thermo Scientific) was added to the final concentration of 1 %. Samples were incubated for 20 min at room temperature while rocking. Formaldehyde was quenched with 120 μl of 2 M Glycine stock solution, samples were incubated at room temperature for 15 min while rocking. Samples were spun for 3 min at maximum speed (15,000 RPM) at room temperature. Pellets were washed twice with 500 μl of ice cold phosphate buffered saline (PBS) containing 1/100 dilution of HALT protease and phosphatase inhibitor cocktail (Thermo Scientific). Worm pellets were resuspended in 500 μl Hi-C lysis buffer (10 mM HEPES pH = 8.0, 10 mM NaCl, 0.2 % IGEPAL CA-630, and 1/100 HALT) and incubated on ice for 15 min. The resulting lysate was spun at maximum speed for 5 min at 4 °C. The supernatant was discarded and pellets were washed with 500 μl of ice-cold wash buffer (50 mM Tris-HCl at pH 8.0, 50 mM NaCl, 1 mM EDTA).

Pellets were resuspended in 500 μl Hi-C nuclear lysis buffer (50 mM Hepes pH = 7.3, 150 mM NaCl, 1 % Triton X-100, 1 mM EDTA, 1 % SDS, 0.1 % Sodium deoxycholate) and rotated at 4 °C for 20 min. Samples were spun at maximum speed for 5 min at 4 °C and supernatant was discarded. Pellets were washed twice with 500 μl ice-cold wash buffer (50 mM Tris-HCl at pH 8.0, 50 mM NaCl, 1mM EDTA) and resuspended in the same buffer to a final volume of 250 μl.

In order to solubilize cross-linked chromatin, samples were mixed with 95 μl of 2 % SDS and incubated at 65 °C for 10 min. Suspensions were cooled down to room temperature before they were mixed with 105 μl 25 mM EZ-Link Iodoacetyl-PEG2-Biotin (IPB) (Thermo Fisher Scientific) to biotinylate proteins. After incubating for 1 h at room temperature while rotating, the SDS was neutralized by adding 1.3 ml 1× NEBuffer 2 (New England Biolabs [NEB], Ipswich, MA, USA). Samples were mixed with 225 μl 10 % Triton X-100 to a final concentration of 1 % and incubated for 10 min on ice, followed by 10 min at 37 °C.

5 μl 1 M DTT, 100 μl 10× NEBuffer 2, 415 μl water and 100 μl of DpnII (or AvaII) restriction enzyme (NEB) (10 U/μl) was added to digest the DNA overnight at 37 °C in a total volume of 2530 μl. After digestion, samples were loaded into a Slide-A-Lyzer Dialysis Cassette G2 (Thermo Fisher Scientific) and dialyzed for 4 h at room temperature against 1 L of dialysis buffer (10 mM Tris-HCl at pH 8.0, 1 mM EDTA) to eliminate excess IPB remaining from the biotinylation step. Dialysis buffer was renewed after 3 h.

400 μl MyOne Streptavidin T1 beads (Life Technologies) were washed 3 times with PBS + 0.01 % Tween-20 (PBST) and beads were resuspended in 2 ml PBST. Dialyzed samples were divided into 5 equal aliquots of 500 μl in 1.5 ml Eppendorf Protein LoBind tubes. 400 μl beads were added to each tube and samples were incubated for 30 min at room temperature while rotating. To prevent interference of unbound streptavidin on the beads with later steps (adding biotinylated dCTP) 5 μl neutralized IPB was added to each tube. IPB was neutralized by adding an equimolar amount of 2-mercaptoethanol. Samples were incubated for an additional 15 min at room temperature while rotating. Non-biotinylated chromatin and non-cross-linked DNA were removed by washing the magnetic T1 beads once with 600 μl PBST and once with 600 μl wash buffer (10 mM Tris-HCl at pH 8.0, 50 mM NaCl, 0.4 % Triton X-100). Beads were resuspended in 100 μl of the same wash buffer. Restriction enzyme (DpnII or AvaII) generated 5’ overhangs were filled in by adding 63 μl water, 1 μl 1 M MgCl, 10 μl 10× NEBuffer 2, 0.7 μl 10 mM dATP, 0.7 μl 10 mM dTTP, 0.7 μl 10 mM 2’-Deoxyguanosine-5’-O-(1-thiotriphosphate), sodium salt, Sp-isomer (Axxora, San Diego, CA, USA), 15 μl 0.4 mM Biotin-14-dCTP (Life Technologies), 4 μl 10 % Triton X-100 and 5 μl 5 U/μl DNA Polymerase I, Large (Klenow) Fragment (NEB).

Samples were incubated for 40 min at room temperature while rotating. Reaction was stopped by adding 5 μl 0.5 M EDTA to the suspension. After 2 min of incubation at room temperature while rotating, beads were washed twice with 600 μl buffer (50 mM Tris-HCl at pH 7.4, 0.4 % Triton X-100, 0.1 mM EDTA) and resuspended in 500 μl of the same buffer. Each sample was transferred into a 15 ml centrifuge tube. For blunt-end ligation under dilute conditions 500 μl sample was mixed with 4 ml water, 250 μl 10× Ligase Buffer (NEB), 100 μl 1 M Tris-HCl at pH 7.4, 90 μl 20 % Triton X-100, 50 μl 100× BSA and 2 μl 2000 U/μl T4 DNA Ligase (NEB), and incubated overnight at 16 °C.

The overnight ligation reaction was stopped by adding 200 μl 0.5M EDTA to each tube. The magnetic T1 beads were collected on the wall of the tube using a magnet and the solution was aspirated out of the tube. The beads were resuspended in 400 μl extraction buffer (50 mM Tris-HCl at pH 8.0, 0.2 % SDS, 1 mM EDTA, 500 mM NaCl) and the mix was transferred into a Eppendorf Protein LoBind microcentrifuge tube. Samples were treated with 5 μl RNase A (20 mg/ml) (Life Technologies) for 45 min at 37 °C and with 20 μl Proteinase K (20 mg/ml) (NEB) overnight at 45 °C.

After overnight incubation, an additional 5 μl Proteinase K were added and samples were incubated for another 2 h at 45 °C. Beads were collected on the wall of the tube and DNA was extracted from the supernatant once with an equal volume of phenol:chloroform (1:1) and once with an equal volume of chloroform. The aqueous phase was mixed with 100 μl of 5 M Ammonium Acetate and 4 μl of 15 mg/ml of glycoblue (Ambion). DNA was precipitated by adding 2.5 volumes of pure ethanol. Precipitated DNA was pelleted by centrifugation at maximal speed (15000RPM) for 30 min at 4 °C. Pellets were washed with ice-cold 70 % ethanol and resuspended in 20 μl 10 mM Tris-HCl at pH 8.0.

100 ng of DNA were subjected to 5 μl Nextera tagmentase (TDE1) at 55 °C for 10 min and purified and concentrated using the DNA Clean & Concentrator™-5 kit (Zymo Research). The purified DNA was eluted in 50 μl of elution buffer. 10 μl of MyOne Streptavidin C1 magnetic beads (Invitrogen) were washed twice with 500 μl 1× Bind & Wash (B&W) buffer (5 mM Tris-HCl at pH 7.4, 0.5 mM EDTA, 1 M NaCl) and resuspended in 50 μl 2× B&W buffer. The purified DNA sample and the C1 beads were mixed and incubated at room temperature for 30 min. The beads were washed once with 500 μl 1× B&W buffer with 0.1 % Triton, once with 500 μl 10 mM Tris-HCl at pH = 8.0 and were resuspended in 20 μl of Resuspension buffer (Nextera DNA Library Prep Kit, Illumina [http://www.illumina.com/products/nextera_dna_library_prep_kit.html]).

The solution with the beads was used directly for PCR amplification according to the Nextera DNA Library Kit protocol, with 12 rounds of PCR. Size selected fragments from a 1 % agarose gel (~500 base pairs) were used for sequencing. The libraries were sequenced using various paired end read lengths with MiSeq, NextSeq and HiSeq Illumina instruments.

### Computational pipeline/methods/procedures

Read pairs obtained by massive parallel sequencing were aligned to the *C. elegans* reference genome (ce10) using an iterative mapping approach utilizing ICE software as described in [38] and available for download from [https://bitbucket.org/mirnylab/hiclib]. As a starting point for this approach, a first portion of the read is aligned to the reference genome, while the read is truncated to a certain length; subsequently the algorithm aggregates alignments over increasing truncation lengths. The mapping utilizes Bowtie2-2.2.5 software [39] and allows detection of several types of double sided mapped reads.

The genome was binned into 50KB non-overlapping intervals, and uniquely mapped read pairs were used to create matrices of contacts between 50KB intervals. Binned data for observed contacts between any two regions was normalized to products of sequencing coverage for the two regions, using HOMER v4.7 software [40].

### Data

Raw read counts and bulk read properties from each individual experiment are presented in Tables 2, 3, 4, 5 and 6, which also provides NCBI-GEO accession numbers. All data are available at the NCBI Gene Expression Omnibus (GEO) repository, accession number GSE76930.

**Table 2:** Mapping statistics for different experimental replicates

Technology	HiSeq 100X2	HiSeq 100X2	HiSeq 100X2	HiSeq 100X2
Sample	N2 L1 Fed AvaII	N2 L1 Fed DpnII	N2 L1 Starved AvaII	N2 L1 Starved DpnII
GEO accession	GSM2041035- SRR3105470	GSM2041036- SRR3105472	GSM2041033- SRR3105465	GSM2041034- SRR3105468
Total number of reads	45,346,483	50,098,648	12,074,391	31,699,338
Both sides aligned	29,802,885	29,416,663	8,325,030	22,664,227
Concordant Pairs	20,376,818	16,485,927	4,352,019	12,630,225
intra-chromosomal Pairs	3,943,229	5,728,073	2,103,366	6,332,935
inter-chromosomal Pairs	1,712,801	3,814,807	559,830	2,321,074
Hi-C valid pairs	5,656,030	9,542,880	2,663,196	8,654,009
% Hi-C valid pairs	19	32.4	32	38.2

**Table 3:** Mapping statistics for different experimental replicates

Technology	NextSeq 38X2	NextSeq 38X2	NextSeq 38X2	NextSeq 38X2
Sample	N2 Adults AvaII	N2 Adults DpnII	glp-1 Adults AvaII	glp-1 Adults DpnII
GEO accession	GSM2041037 - SRR3105473	GSM2041038 - SRR3105476	GSM2041039 - SRR3105478	GSM2041040 – SRR3105481
Total number of reads	92,039,660	101,295,678	67,052,017	195,829,257
Both sides aligned	50,361,629	54,717,285	34,869,674	101,403,196
Concordant Pairs	15,870,773	20,572,866	11,655,151	30,231,656
intra-chromosomal Pairs	3,131,465	14,303,981	4,519,524	17,089,727
inter-chromosomal Pairs	1,207,782	4,628,515	1,182,249	5,294,659
Hi-C valid pairs	4,339,247	18,932,496	5,701,773	22,384,386
% Hi-C valid pairs	8.6	34.6	16.4	22.1

**Table 4:** Mapping statistics for different experimental replicates

Technology	MiSeq 78X2	MiSeq 78X2	MiSeq 78X2	MiSeq 78X2
Sample	N2 Adults AvaII	N2 Adults DpnII	glp-1 Adults AvaII	glp-1 Adults DpnII
GEO accession	GSM2041037- SRR3105474	GSM2041038 - SRR3105477	GSM2041039 - SRR3105479	GSM2041040 –SRR3105482
Total number of reads	6,209,078	6,763,705	5,447,839	9,770,754
Both sides aligned	4,206,958	4,879,863	3,807,291	6,697,162
Concordant Pairs	2,888,881	2,439,233	2,284,399	3,739,874
intra-chromosomal Pairs	642,277	1,718,469	911,315	1,863,649
inter-chromosomal Pairs	223,318	553,223	223,826	573,671
Hi-C valid pairs	865,595	2,271,692	1,135,141	2,437,320
% Hi-C valid pairs	20.6	46.6	29.8	36.4

**Table 5:** Mapping statistics for different experimental replicates

Technology	MiSeq 250X2	MiSeq 250X2	MiSeq 250X2	MiSeq 250X2
Sample	N2 L1 Fed AvaII	N2 L1 Fed DpnII	N2 L1 Starved AvaII	N2 L1 Starved DpnII
GEO accession	GSM2041035- SRR3105469	GSM2041036- SRR3105471	GSM2041033 - SRR3105465	GSM2041034- SRR3105467
Total number of reads	5,058,249	4,682,335	6,887,612	3,716,627
Both sides aligned	2,438,224	1,576,630	3,552,524	1,987,126
Concordant Pairs	1,928,195	1,094,704	2,506,096	1,159,604
intra-chromosomal Pairs	323,168	278,034	677,872	583,798
inter-chromosomal Pairs	143,311	193,579	210,296	222,675
Hi-C valid pairs	466,479	471,613	888,168	806,473
% Hi-C valid pairs	19.1	30	25	40.6

**Table 6:** Mapping statistics for different experimental replicates

Technology	NextSeq 151X1	NextSeq 151X1
Sample	N2 Adults DpnII	glp-1 Adults DpnII
GEO accession	GSM2041038 – SRR3105475	GSM2041040 - SRR3105480
Total number of reads	36,806,738	55,610,584
Both sides aligned	10,674,057	5,964,566
Concordant Pairs	4,145,075	1,826,523
intra-chromosomal Pairs	2,795,635	1,649,699
inter-chromosomal Pairs	1,690,332	1,877,419
Hi-C valid pairs	4,485,967	3,527,118
% Hi-C valid pairs	42	59.1

## Additional files

Additional file 1: Figure S1. Correlation between different experiments. The genome was divided into 50KB non-overlapping segments and chromatin contact matrices were generated. In order to evaluate the correlation between experiments we calculated the normalized levels of contacts for all matrix locations (dividing by the total number of contacts detected). Then we calculated correlations between normalized values of contacts of different experiments. x-axis and y-axis stand for normalized contacts values calculated for different experiments. The correlation analysis was performed between experiments done with four *C. elegans* populations:(i) N2 L1 starved animals, (ii) N2 L1 fed animals, (iii) N2 young adult animals,(iv) *glp-1(e2141ts)III* [33, 35] young adults (populations grown at the permissive temperature (16 °C) to L1 stage, then shifted to the restrictive temperature (23 °C) [36] to adulthood). Analysis was performed with DpnII and AvaII restriction enzymes as noted. Any contacts between any location on chromosome I and region containing rRNA on chromosome I (the bin 15,050,000-end of chromosome I) are colored in green. (PDF 9816 kb) Additional file 2: Figure S2. Correlation between N2 DpnII experimental data and data from Crane et al. [22]. The 50KB chromatin contact matrix constructed using N2 young adults treated with DpnII restriction enzyme data (GSM2041038- SRR3105476) was compared with 50KB resolution chromatin contacts matrix constructed using the Crane et al. data (GSM1556154 - SRR1665087) from [22]. The total number of paired-ended 37X2 reads in our dataset was 88,466,514, while Crane et al. provide a total of 115,983,178 paired-ended 100X2 reads was. In order to build the chromatin contacts matrix we used the ICE pipeline [38] iterative mapping implementation from [https://bitbucket.org/mirnylab/hiclib], starting from 21nt up to 37nt in increments of 8. The number of detected Hi-C valid pairs in our dataset was 18,779,498 , consisting of 4,542,078 inter-chromosomal contacts and 14,237,420 intra-chromosomal contacts. In Crane’s dataset the number of valid Hi-C pairs was 59,200,047, consisting of 6,457,271 inter-chromosomal contacts and 52,742,776 intra-chromosomal contacts. Similarly to Additional file 1: Figure S1, any contacts between any location on chromosome I and region containing rRNA on chromosome I (the bin 15,050,000-end of chromosome I) are colored in green. Contacts between genomic loci in adjacent regions (up to 100KB apart are colored in purple). Versions of software used for analysis are as follows: Bowtie2-2.2.6 [39], and mirnylib/hiclib [https://bitbucket.org/mirnylab/hiclib] downloaded on December 1, 2015. Slight differences in aligned read counts from Tables 2 and 3 reflect updates in alignment software in the concerted package compared to the legacy versions used in Tables 2 and 3. (PDF 1132 kb) Additional file 3: Figure S3. Comparison between RTCC experimental data (this work; black lines), Hi-C data from Crane et al. (2015; magenta lines); and relative representation in anti-LEM2 ChIP-chip data [28]. The curves were obtained by running the ICE pipeline [38] on our N2 dataset (N2 DpnII GSM2041038- SRR3105476) and on Crane et al. dataset (GSM1556154 - SRR1665087), as implemented in [https://bitbucket.org/mirnylab/hiclib], downloaded on Dec 1, 2015. To obtain the first Eigen Vector values, representing compartments along the chromosome axis, we have followed the tutorial from [https://bitbucket.org/mirnylab/hiclib], using the binnedData class function doEig(numPCs = 1). To inspect the correlation to LEM-2 binding compartments we added LEM-2 binding data [28] (MA2C normalized log_2_ ratio of ChIP signal over control), lifted from the ce4 genome assembly to the ce10 assembly, and averaged in 50KB bins. (PDF 46 kb)

## Abbreviations

3C: chromosome conformation capture
GEO: gene expression omnibus
PATC: periodic A/T cluster
RTCC: rapid tethered conformation capture
TCC: tethered conformation capture
