# Supplementary material for: A streamlined tethered chromosome conformation capture protocol
Source: BMC Genomics. 2016 Apr 1;17:274. doi: 10.1186/s12864-016-2596-3 (PMC4818521; doi:10.1186/s12864-016-2596-3)
Supplement: Additional file 1: Figure S1. — Correlation between different experiments. The genome was divided into 50KB non-overlapping segments and chromatin contact matrices were generated. In order to evaluate the correlation between experiments we calculated the normalized levels of contacts for all matrix locations (dividing by the total number of contacts detected). Then we calculated correlations between normalized values of contacts of different experiments. x-axis and y-axis stand for normalized contacts values calculated for different experiments. The correlation analysis was performed between experiments done with four C. elegans populations:(i) N2 L1 starved animals, (ii) N2 L1 fed animals, (iii) N2 young adult animals,(iv) glp-1(e2141ts)III [33, 35] young adults (populations grown at the permissive temperature (16 °C) to L1 stage, then shifted to the restrictive temperature (23 °C) [36] to adulthood). Analysis was performed with DpnII and AvaII restriction enzymes as noted. Any contacts between any location on chromosome I and region containing rRNA on chromosome I (the bin 15,050,000-end of chromosome I) are colored in green. (PDF 9816 kb) [file 12864_2016_2596_MOESM1_ESM.pdf]

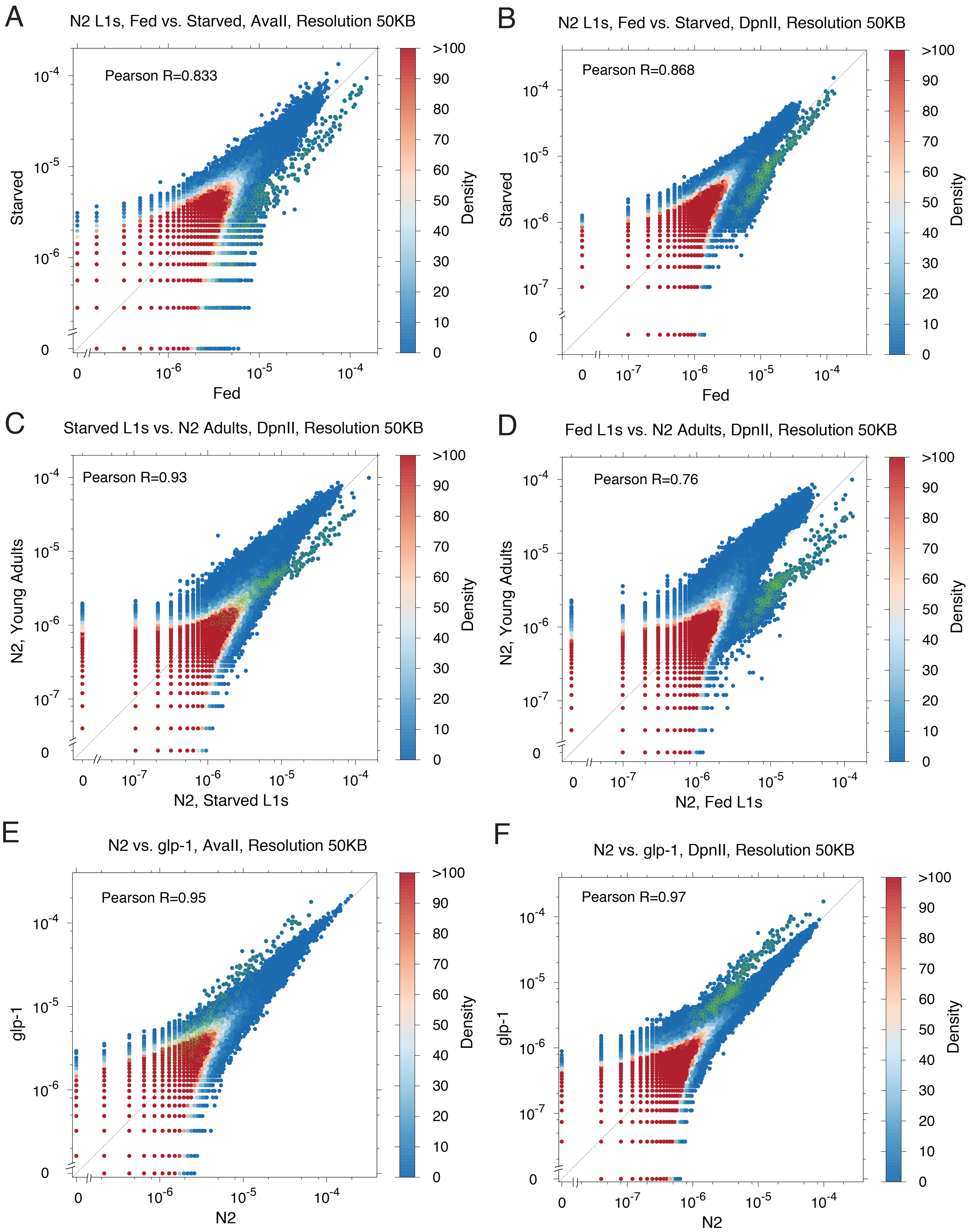
A
N2 L1s, Fed vs. Starved, AvaII, Resolution 50KB
Pearson R=0.833
Starved
Fed
Density
B
N2 L1s, Fed vs. Starved, DpnII, Resolution 50KB
Pearson R=0.868
Starved
Fed
Density
C
Starved L1s vs. N2 Adults, DpnII, Resolution 50KB
Pearson R=0.93
N2, Young Adults
N2, Starved L1s
Density
D
Fed L1s vs. N2 Adults, DpnII, Resolution 50KB
Pearson R=0.76
N2, Young Adults
N2, Fed L1s
Density
E
N2 vs. glp-1, AvaII, Resolution 50KB
Pearson R=0.95
glp-1
N2
Density
F
N2 vs. glp-1, DpnII, Resolution 50KB
Pearson R=0.97
glp-1
N2
Density
>100
90
80
70
60
50
40
30
20
10
0
