# Supplementary figures and images for: A streamlined tethered chromosome conformation capture protocol
Source: BMC Genomics. 2016 Apr 1;17:274. doi: 10.1186/s12864-016-2596-3 (PMC4818521; doi:10.1186/s12864-016-2596-3)

Comparison with E. Crane et. al data, N2 (DpnII), Resolution 50KB

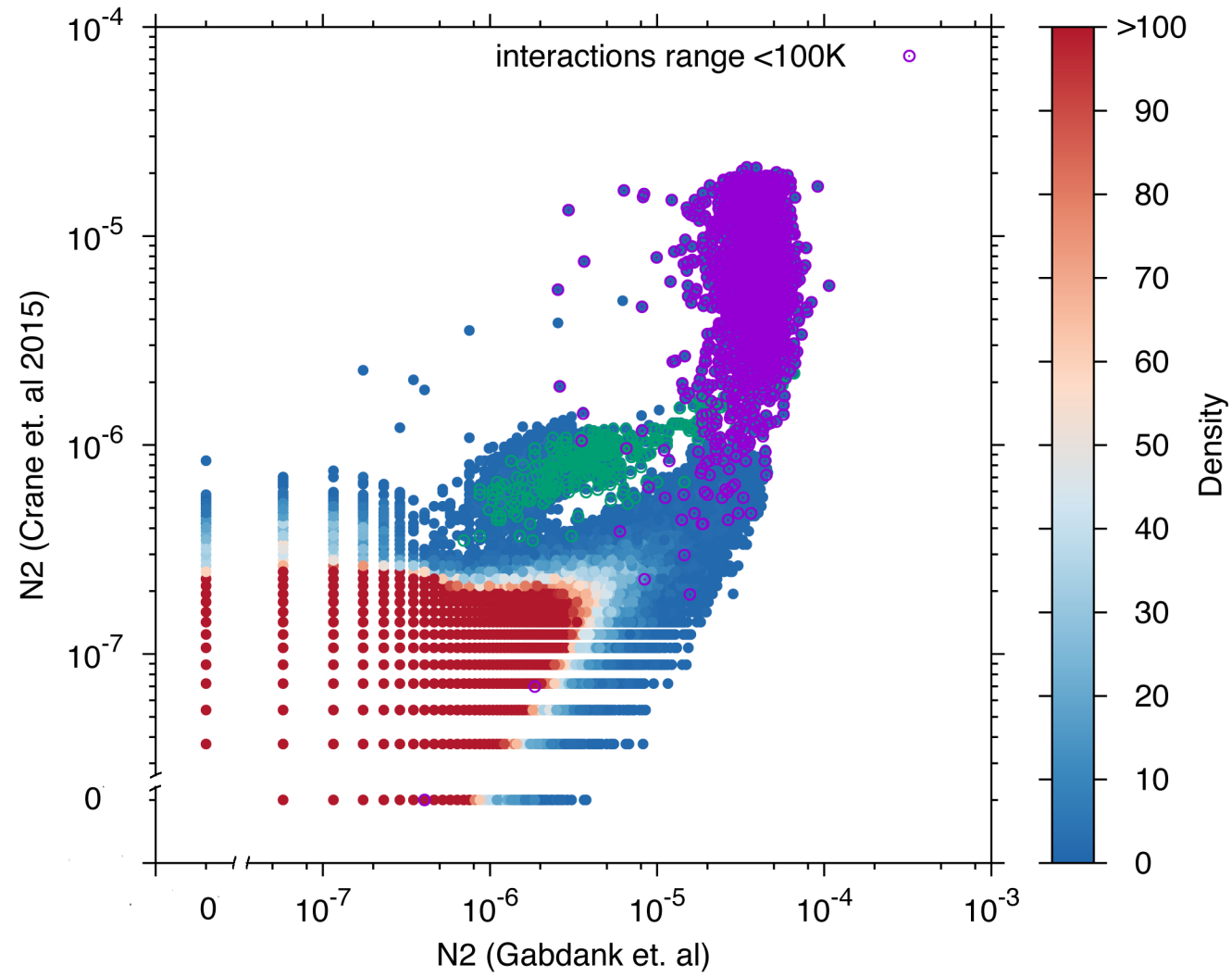

Supplement: Additional file 2: Figure S2. — Correlation between N2 DpnII experimental data and data from Crane et al. [22]. The 50KB chromatin contact matrix constructed using N2 young adults treated with DpnII restriction enzyme data (GSM2041038- SRR3105476) was compared with 50KB resolution chromatin contacts matrix constructed using the Crane et al. data (GSM1556154 - SRR1665087) from [22]. The total number of paired-ended 37X2 reads in our dataset was 88,466,514, while Crane et al. provide a total of 115,983,178 paired-ended 100X2 reads was. In order to build the chromatin contacts matrix we used the ICE pipeline [38] iterative mapping implementation from [https://bitbucket.org/mirnylab/hiclib], starting from 21nt up to 37nt in increments of 8. The number of detected Hi-C valid pairs in our dataset was 18,779,498 , consisting of 4,542,078 inter-chromosomal contacts and 14,237,420 intra-chromosomal contacts. In Crane’s dataset the number of valid Hi-C pairs was 59,200,047, consisting of 6,457,271 inter-chromosomal contacts and 52,742,776 intra-chromosomal contacts. Similarly to Additional file 1: Figure S1, any contacts between any location on chromosome I and region containing rRNA on chromosome I (the bin 15,050,000-end of chromosome I) are colored in green. Contacts between genomic loci in adjacent regions (up to 100KB apart are colored in purple). Versions of software used for analysis are as follows: Bowtie2-2.2.6 [39], and mirnylib/hiclib [https://bitbucket.org/mirnylab/hiclib] downloaded on December 1, 2015. Slight differences in aligned read counts from Tables 2 and 3 reflect updates in alignment software in the concerted package compared to the legacy versions used in Tables 2 and 3. (PDF 1132 kb) [file 12864_2016_2596_MOESM2_ESM.pdf]

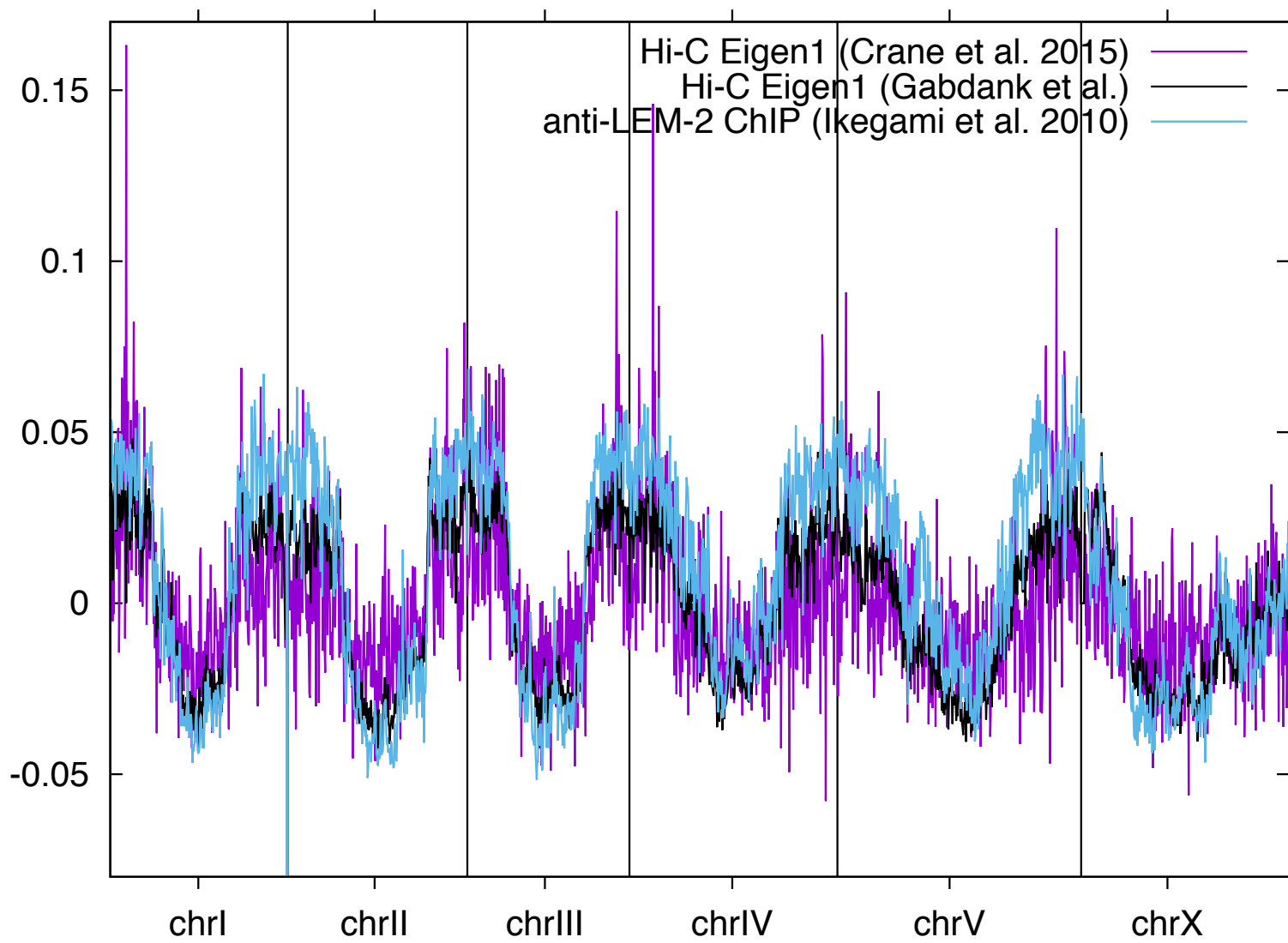

Supplement: Additional file 3: Figure S3. — Comparison between RTCC experimental data (this work; black lines), Hi-C data from Crane et al. (2015; magenta lines); and relative representation in anti-LEM2 ChIP-chip data [28]. The curves were obtained by running the ICE pipeline [38] on our N2 dataset (N2 DpnII GSM2041038- SRR3105476) and on Crane et al. dataset (GSM1556154 - SRR1665087), as implemented in [https://bitbucket.org/mirnylab/hiclib], downloaded on Dec 1, 2015. To obtain the first Eigen Vector values, representing compartments along the chromosome axis, we have followed the tutorial from [https://bitbucket.org/mirnylab/hiclib], using the binnedData class function doEig(numPCs = 1). To inspect the correlation to LEM-2 binding compartments we added LEM-2 binding data [28] (MA2C normalized log2 ratio of ChIP signal over control), lifted from the ce4 genome assembly to the ce10 assembly, and averaged in 50KB bins. (PDF 46 kb) [file 12864_2016_2596_MOESM3_ESM.pdf]
